# Haematological and Biochemical Parameters during the Laying Period in Common Pheasant Hens Housed in Enhanced Cages

**DOI:** 10.1155/2014/364602

**Published:** 2014-07-09

**Authors:** Petra Hrabčáková, Eva Voslářová, Iveta Bedáňová, Vladimíra Pištěková, Jan Chloupek, Vladimír Večerek

**Affiliations:** ^1^Department of Veterinary Public Health and Animal Welfare, Faculty of Veterinary Hygiene and Ecology, University of Veterinary and Pharmaceutical Sciences Brno, Palackeho Třída 1/3, 612 42 Brno, Czech Republic; ^2^Department of Animal Husbandry and Animal Hygiene, Faculty of Veterinary Hygiene and Ecology, University of Veterinary and Pharmaceutical Sciences Brno, Palackeho Třída 1/3, 612 42 Brno, Czech Republic

## Abstract

The development of selected haematological and biochemical parameters during the laying period was monitored in common pheasant hens housed in an enhanced cage system. The cages were enhanced by the addition of two perches and a shelter formed by strips of cloth hanging in the corner of the cage. The results showed significant changes in the haematological and biochemical parameters monitored during egg laying. At the time when laying capacity approached a maximum, a decrease was observed (*P* < 0.05) in haematocrit, erythrocytes, and haemoglobin values, whereas monocytes, eosinophils, the heterophil/lymphocyte ratio, phosphorus, and calcium exhibited an increase (*P* < 0.05). At the end of the laying period, an increase (*P* < 0.05) was recorded in the count of leukocytes, heterophils, lymphocytes and basophils, the heterophil to lymphocyte ratio, and the concentrations of aspartate aminotransferase, cholesterol, phosphorus, and calcium, whereas lower values (*P* < 0.05) were recorded for haematocrit and plasma total protein in comparison with the values of the indicators at the beginning of the laying period. The results provide new information about dynamic changes in selected haematological and biochemical parameters in clinically healthy common pheasant hens during the laying period.

## 1. Introduction

The common pheasant is widespread throughout the world in introduced feral populations and in farm operations. It is native to Russia and has been widely introduced elsewhere as a game bird [1]. In the past, game was associated primarily with hunting and shooting, whereas today it is a freely available commodity of increasing popularity [2]. Feathered game, including the common pheasant (*Phasianus colchicus*), also has its place in the market.

Pheasants are usually kept in large flocks [3], in systems with external aviaries, harems, and colonies [4], in conventional cage systems [5], or more recently in enhanced cage systems [6].

Throughout the farm cycle, pheasants are usually bred from the age of one to four years (in some cases up to the age of seven), at a sex ratio from 1 : 5 to 1 : 7, male : female. Hen pheasants for breeding lay an average of 50 to 70 eggs during the reproductive season. As reported by González-Redondo and García-Domínguez [4] in Spain this season usually lasts from March to April and sometimes even until June or July.

Biochemical parameters may be used as physiological indicators in birds. These values are influenced by species, age, sex, season, geographic region, nutrition, and physiological condition [7–9]. They are also indicators of the health of birds housed in cage systems. Only a few studies have been published focusing on biochemical parameters in pheasants [10–15].

Knowledge of bird haematology is also a useful diagnostic tool in veterinary medicine, and these values can be used as physiological indicators. Haematological values are commonly used as an indicator of health in birds housed in cage systems to detect stress caused by various factors, such as environmental, nutritional, and pathological aspects [3, 16]. Only a few studies have been published to date focusing on the use of haematology indices in pheasants [3, 10–12].

It is important to monitor the health of pheasants in captivity as they relate to the welfare of the birds and the quality of meat produced. Despite the large number of publications that deal with the reference values of haematological and biochemical indicators in poultry, there is a lack of knowledge about these parameters in feathered game, including the common pheasant. In particular, there is no information about dynamic changes and the haematological and biochemical profile of pheasant blood during laying.

The main purpose of this study was to investigate changes in the values of selected haematological and biochemical parameters in common pheasant hens housed in enhanced cages during the laying period.

## 2. Materials and Methods

### 2.1. The Birds and Their Treatment

The experiment was performed during the period April–June 2011 on approximately one-year-old (average body weight of 0.85 kg) common pheasant hens (*Phasianus colchicus*). The birds were housed in external aviaries at the rearing facility prior to the experiment. The number of birds in the pheasantry of University of Veterinary and Pharmaceutical Sciences Brno in Jinačovice was approximately 900 (parent flock). The pheasant hens were housed in a two-tiered cage battery from the beginning of the laying period. Each cage was enhanced with two perches and a place to hide created by strips of cloth hung in one corner of the cage, separating this corner from the rest of the cage. There was one breeding group in each cage, consisting of one cock and five hens. Each laying cage was equipped with a wire floor, five automatic nipple drinkers, and a trough feeder located on the front wall of the cage, with manual distribution of the feed. The dimensions of the cage were as follows: 200 cm length, 85 cm depth, 58 cm back height, and 70 cm front height. The floor was sloped towards the front wall to enable the collection of laid eggs. The pheasants were fed with a BZN pelleted feed mixture (ADW Agro a.s., Krahulov, Czech Republic). The cages were kept under natural light only (daylight), with no artificial lighting. All pheasants were ringed; that is, an individually numbered plastic tag was attached to the leg of each bird.

At the beginning of the laying period, 15 pheasant hens from 15 randomly selected cages (each hen from a different cage) were selected at random and sampled for biochemical and haematological blood analysis. Subsequently, the same hens were sampled after 6 and 12 weeks of the laying period.

### 2.2. Blood Sampling

Blood samples (3 mL) for haematological and biochemical examination were taken from the* vena basilica* of the left wing; these were collected using syringe-needle assemblies that had been flushed with heparin (15 *μ*L of heparin per 3 mL of blood). The samples were collected within 1 minute of capture to ensure that the levels of the monitored parameters were not affected by any stress induced by presampling handling [17]. Blood sampling was always performed at the same time of day (9.00 a.m.) and did not exceed one hour in duration. The heparinised blood was immediately centrifuged at 837 ×g at 4°C for 10 minutes, and plasma samples were stored at −80°C in Eppendorf test tubes until the analyses were performed. The samples for haematological examination were collected in tubes with EDTA and analysed immediately.

### 2.3. Biochemical Examinations

Selected plasma biochemical parameters (total protein, albumin, cholesterol, glucose, calcium, phosphorus, uric acid, aspartate aminotransferase, lactate dehydrogenase, and lactate) were measured on the principle of photometric detection in a KONELAB 20i biochemical analyser using commercial test kits (Biovendor-Laboratorni medicina a.s., Brno, Czech Republic).

### 2.4. Haematological Examinations

Total erythrocyte and leukocyte counts were measured using the dilution flask method and the counting of corpuscles using a Bürker chamber [18]. The diluent was a Natt-Herrick solution [14] and the dilution rate was 1 : 199 (a mixture of 25 *μ*L blood and 4.975 *μ*L Natt-Herrick solution was prepared). Haemoglobin levels were recorded photometrically using a SPECOL-11 photometer and Drabkin's solution [19] at a wavelength of 540 nm, and haematocrit values were determined by means of the microhematocrit technique according to Janetzki [20]. The proportions of individual leukocyte types (in 200 cells) were computed using a light microscope with an immersion lens [18]. Blood smears were prepared using a coverslip technique and were air-dried. The Pappenheim method of biphasic staining with May-Grunwald and Giemsa-Romanowsky stains was used [21, 22]. The type of blood cells was determined according to Lucas and Jamroz [23].

### 2.5. Statistical Analysis

The results were analysed using the UNISTAT 5.1 (Unistad Ltd., GB) statistical package. The normality and homogeneity of variances were checked for all variables tested by means of a Shapiro-Wilk test and a Bartlett-Box test [24]. In the case of nonnormal data, logarithmic or square root transformations were used for the analysis of variance, though actual mean values are presented in the tables. Because the same birds were measured on each day of sampling over the length of the experiment, the data was subjected to repeated ANOVA measures using the general linear model procedure with a “day of sampling” factor with 3 levels (day 1, day 42, and day 84) among the birds in a randomised block design. There were *n* = 15 pheasants (blocks) in the experiment. Where the effect was statistically significant, a Tukey-HSD test was performed as a* post hoc* test for pairwise comparisons of means. A *P* value of <0.05 was considered significant.

## 3. Results and Discussion

Haematological testing is one of the methods that can help detect certain changes in health that may not be apparent from physical examination, but which affect, for example, the condition of the birds [25–27]. Our study is concentrated on the three months during the laying period, which can be considered a significant factor affecting the values of the haematological parameters of laying hens during this period. The haematological profile of pheasant hens during the laying period is shown in Table 1.

Haematocrit values were lower during the laying period. A highly statistically significant decrease in haematocrit values in laying hens was recorded in week 6 (*P* < 0.001) and in week 12 (*P* < 0.001) compared with the haematocrit values at the beginning of laying.

Our results show a decrease in the red blood cell count and haemoglobin content relative to the approach of the peak laying period. The lowest average values of the total erythrocyte count (2.66 ± 0.09 10^12^/L) and haemoglobin content (108.0 ± 2.9 g/L) were determined in week 6 of laying, which is roughly the period when the rate of laying of the common pheasant hen reaches its maximum, although the academic sources differ from this figure (e.g., Gibes et al. [28], Woodard and Snyder [29], and Krystianiak et al. [30]). A drop in the total number of erythrocytes (*P* < 0.001) and haemoglobin (*P* = 0.0356) in week 6 was followed by a significant increase in both indicators (*P* < 0.001) in week 12. Similarly to Straková et al. [31], in laying hens, we assume that the laying period for pheasants represents a significant amount of metabolic stress that is reflected in a reduced number of red blood cells.

In our study, the common pheasant hens showed an increase in the number of leukocytes (*P* = 0.007) and heterophils (*P* = 0.0248) during the laying period. The average numbers of white blood cells found in this study were within the normal values of the usual range (15 to 30 × 10^3^ cells/mm^3^) listed for the majority of bird species [13, 32, 33]. Despite heterophils being the most abundant leukocyte in the peripheral blood of most species of birds, the major circulating leukocytes in pheasants, regardless of age, are lymphocytes [11, 34].

A significant increase was recorded between week 1 and week 12 of laying in lymphocytes (*P* = 0.0134), basophils (*P* = 0.0083), and the heterophil/lymphocyte ratio (HLR) (*P* = 0.0324). Like Keçeci and Çöl [13], we recorded lymphocytes as they are the main type of white blood cells in the blood of pheasants. This predominance of lymphocytes is also confirmed by Schmidt et al. [11] in pheasants. The HLR is a good indicator of stress in chickens [35]. The difference between the levels in adult females, males, and young pheasants may indicate physiological stress, probably caused by the laying cycle in adult females [12, 36]. According to the results recorded by Schmidt et al. [12], adult female common pheasants had higher absolute values of basophils than adult males and young pheasants. Since the HLR was higher in adult females, it appears that the increased number of basophils indicates the same state of physiological stress [36]. An increase in the HLR in chickens in conventional cages in the study by Shini [37] showed that these chickens were subjected to an optimum degree of stress, as shown by an H/L ratio of 0.58. A significant increase was recorded between weeks 1 and 6 of laying for eosinophils (*P* = 0.0066) and monocytes (*P* = 0.0054). A decrease was then seen in the level of monocytes in week 12 of laying (*P* = 0.0490).

Our knowledge of biochemical parameters in the common pheasant is still incomplete [14]. To date, only a few studies of the values of biochemical parameters in pheasants have been published; however, none of these focus on changes in the biochemical parameters during laying.

Pavlík et al. [38] demonstrated the effect (*P* < 0.05 and *P* < 0.01) of age and/or the phase of the laying cycle on the concentrations of all minerals. The mineral concentration of blood plasma [38] and biochemical values may be affected by many factors, such as the rate of laying and energy requirements [39]. The relationship between the phase of the laying cycle and the values of selected biochemical indicators in common pheasant hens resulting from our study is presented in Table 2.

Our results show a decrease in the protein content of blood plasma during the laying period of twelve weeks (*P* = 0.0082). A statistically significant drop in blood plasma proteins (*P* = 0.0011) in common pheasant hens was seen, particularly between weeks 6 and 12 of laying. The values for the total protein content in the majority of birds fall within the range of 30–50 g/L [10, 14, 40–44]. Significant differences in serum total protein and albumin among males and females during breeding season could be due to the egg production that may affect the concentration of the blood proteins [45, 46].

The levels of cholesterol (*P* = 0.0188), phosphorus (*P* = 0.0002), and calcium (*P* < 0.001) showed a statistically significant increase during the laying period in our study. The standard range of values of cholesterol in domestic fowl is 3.55 to 10.25 mmol/L [31, 47]. The cholesterol level may increase significantly during* vitellogenesis* and egg formation in birds [48]. As has already been published, the wide variation in cholesterol and triglyceride levels between species of birds may be due to the circadian rhythms, the effects of diet [48–50], gender, and age [51, 52]. Like Suchý et al. [39], we assume that the increase in the cholesterol level was due to increased biosynthesis and accumulation in the egg yolk. Similarly, the greater release of calcium into the blood of laying hens is closely related to its accumulation in the shell [39].

The concentration of glucose (*P* = 0.0114) and uric acid (*P* = 0.0436) showed a statistically significant increase in week 12 as compared with the concentration in week 6 of the laying period. Normal levels of glucose concentration in the majority of birds are between 200 and 500 mg/dL (11.1–27.8 mmol/L) [53]. The glucose values in the present study were in the normal range as reported in pheasants by Llloyd and Gibson [10] (19.5 mmol/L) and Suchý et al. [44] (20.08 mmol/L) but higher than those reported in pheasants by Nazifi et al. [14] (12.6 mmol/L in females). The uric acid concentration is affected by factors such as species, age, and diet [54].

As blood plasma enzymes are characterised by an extremely broad spectrum of activity, the interpretation of fluctuations in these enzymes is difficult. There is little information about the values of AST and LDH in pheasants with which we could compare our results. In our study, there was no significant change in the levels of ALB and LDH or the concentration of lactate during the laying period. There is also no published data detailing the dynamics of lactate in the blood plasma of pheasant hens. The published data is limited merely to the research of acute stress [17, 55]. A significant increase (*P* = 0.0189) in the concentration of AST was found during the laying period in common pheasant hens. According to Schmidt et al. [56], who studied biochemical parameters in turkeys, certain physiological changes in the metabolism of female birds may occur as a consequence of the laying of eggs. AST values vary, depending on age, to differing degrees in different species [56]. According to Schmidt et al. [45, 46] the egg production may affect the concentration of albumin in pheasants during the breeding season.

## 4. Conclusion

This study describes pattern of development of selected haematological and biochemical parameters in common pheasant hens housed in enhanced cage systems during the laying period. As in poultry, knowledge of the development of dynamic changes in haematological and biochemical parameters is important for the interpretation of physiological processes in the pheasant. At the time when the laying capacity approached a maximum, a fall was recorded in haematocrit, erythrocytes, and haemoglobin values, whereas monocytes, eosinophils, the heterophil/lymphocyte ratio, phosphorus, and calcium increased. At the end of the laying period, an increase was measured in the count of leukocytes, heterophils, lymphocytes and basophils, the heterophil to lymphocyte ratio, and the concentrations of aspartate aminotransferase, cholesterol, phosphorus, and calcium, whereas lower values were recorded for haematocrit and plasma total protein in comparison with the values of the given indicators at the beginning of the laying period. The results provide new information about dynamic changes in selected haematological and biochemical parameters in clinically healthy common pheasant hens during the laying period.

## Figures and Tables

**Table 1:** Haematological parameters in pheasant hens (*n* = 15/group) housed in an enhanced cage system during the laying period (means ± SEM).

Haematological parameters	Sampling day (day of laying period)		
	Day 1	Day 42	Day 84
Haematocrit (L/L)	0.38 ± 0.01^a^	0.33 ± 0.01^b^	0.33 ± 0.01^b^
Haemoglobin (g/L)	118.3 ± 2.7^b^	108.0 ± 2.9^c^	137.3 ± 2.8^a^
Erythrocytes (10^12^/L)	3.51 ± 0.13^a^	2.66 ± 0.09^b^	3.73 ± 0.18^a^
Leukocytes (10^9^/L)	17.0 ± 1.27^b^	21.8 ± 1.65^a,b^	23.6 ± 1.46^a^
Heterophils (10^9^/L)	0.79 ± 0.20^b^	1.56 ± 0.22^a,b^	1.71 ± 0.28^a^
Lymphocytes (10^9^/L)	15.9 ± 1.21^b^	19.8 ± 1.50^a,b^	21.5 ± 1.32^a^
Eosinophils (10^9^/L)	0.08 ± 0.01^b^	0.14 ± 0.02^a^	0.12 ± 0.01^a,b^
Basophils (10^9^/L)	0.08 ± 0.01^b^	0.11 ± 0.01^a,b^	0.16 ± 0.03^a^
Monocytes (10^9^/L)	0.1 ± 0.01^b^	0.17 ± 0.02^a^	0.12 ± 0.01^b^
HLR	0.05 ± 0.01^b^	0.08 ± 0.01^a^	0.08 ± 0.01^a^

^a,b,c^Means within a row with different superscripts differ (*P* < 0.05).

**Table 2:** Selected biochemical parameters in pheasant hens (*n* = 15/group) housed in an enhanced cage system during the laying period (means ± SEM).

Biochemical parameters	Sampling day (day of laying period)		
	Day 1	Day 42	Day 84
Total protein (g/L)	38.6 ± 1.39^a^	41.5 ± 1.55^a^	25.5 ± 4.66^b^
ALB (g/L)	20.4 ± 0.79^a^	20.5 ± 0.67^a^	22.8 ± 1.07^a^
Cholesterol (mmol/L)	6.24 ± 0.67^b^	6.45 ± 0.68^b^	8.35 ± 0.38^a^
Glucose (mmol/L)	20.4 ± 0.26^a,b^	19.6 ± 0.24^b^	20.8 ± 0.33^a^
Calcium (mmol/L)	3.85 ± 0.27^b^	5.22 ± 0.24^a^	5.79 ± 0.35^a^
Phosphorus (mmol/L)	1.49 ± 0.07^b^	1.83 ± 0.09^a^	2.11 ± 0.13^a^
Uric acid (*μ*mol/L)	240.5 ± 28.7^a,b^	217.0 ± 13.3^b^	296.7 ± 23.1^a^
AST (U/L)	293.5 ± 20^b^	336.5 ± 21.2^a^	387.1 ± 27.6^a^
LDH (U/L)	193.4 ± 14.97^a^	225.7 ± 23.95^a^	202.99 ± 17.96^a^
Lactate (mmol/L)	10.9 ± 0.72^a^	10.8 ± 0.57^a^	12.8 ± 0.83^a^

^a,b^Means within a row with different superscripts differ (*P* < 0.05).

## Abbreviations

HLR: Heterophil to lymphocyte ratio
AST: Aspartate aminotransferase
LDH: Lactate dehydrogenase
ALB: Albumin.
